# Glucocorticoids and renal Na^+^ transport: implications for hypertension and salt sensitivity

**DOI:** 10.1113/jphysiol.2013.267609

**Published:** 2014-03-24

**Authors:** Robert W Hunter, Jessica R Ivy, Matthew A Bailey

**Affiliations:** British Heart Foundation Centre for Cardiovascular Science, University of EdinburghEdinburgh, UK

## Abstract

The clinical manifestations of glucocorticoid excess include central obesity, hyperglycaemia, dyslipidaemia, electrolyte abnormalities and hypertension. A century on from Cushing's original case study, these cardinal features are prevalent in industrialized nations. Hypertension is the major modifiable risk factor for cardiovascular and renal disease and reflects underlying abnormalities of Na^+^ homeostasis. Aldosterone is a master regulator of renal Na^+^ transport but here we argue that glucocorticoids are also influential, particularly during moderate excess. The hypothalamic–pituitary–adrenal axis can affect renal Na^+^ homeostasis on multiple levels, systemically by increasing mineralocorticoid synthesis and locally by actions on both the mineralocorticoid and glucocorticoid receptors, both of which are expressed in the kidney. The kidney also expresses both of the 11β-hydroxysteroid dehydrogenase (11βHSD) enzymes. The intrarenal generation of active glucocorticoid by 11βHSD1 stimulates Na^+^ reabsorption; failure to downregulate the enzyme during adaption to high dietary salt causes salt-sensitive hypertension. The deactivation of glucocorticoid by 11βHSD2 underpins the regulatory dominance for Na^+^ transport of mineralocorticoids and defines the ‘aldosterone-sensitive distal nephron’. In summary, glucocorticoids can stimulate renal transport processes conventionally attributed to the renin–angiotensin–aldosterone system. Importantly, Na^+^ and volume homeostasis do not exert negative feedback on the hypothalamic–pituitary–adrenal axis. These actions are therefore clinically relevant and may contribute to the pathogenesis of hypertension in conditions associated with elevated glucocorticoid levels, such as the metabolic syndrome and chronic stress.

## Introduction

It is over 100 years since Harvey Cushing described the clinical consequences of severe glucocorticoid excess (Cushing, 1912) and although this syndrome remains rare, the cardinal features of central obesity, dyslipidaemia, impaired glucose metabolism and hypertension are increasingly prevalent in Western society (Batsis *et al*. 2007). These traits of the ‘metabolic syndrome’ endanger cardiovascular health. Indeed, hypertension is the major modifiable risk factor for both cardiovascular and renal disease and reflects impaired Na^+^ homeostasis and a diminution of the pressure natriuresis mechanisms (Mullins *et al*. 2006). Here, we review recent studies that provide a mechanistic framework for regulation of renal Na^+^ transport by glucocorticoids: two overarching themes are developed. First, that defining glucocorticoid action within the kidney is challenging due to the pleiotropic actions of systemic glucocorticoids. Second, that there are multiple instances where the hypothalamic–pituitary–adrenal axis (HPAA) can influence Na^+^ transport processes that are classically regulated by the renin–angiotensin–aldosterone system (RAAS). Such ‘crosstalk’ may have a physiological context but there is an implicit capacity for aberrant renal Na^+^ transport as the HPAA is not regulated by Na^+^/volume homeostasis.

## Integrated responses to glucocorticoids

The renal response to systemic glucocorticoid administration is well characterized (Table 1) but attempts to resolve these actions into specific tubular and vascular components are confounded by two phenomena: the pleiotropic effects of glucocorticoids and the promiscuity of steroid receptor–ligand interactions (Fig. 1).

**Table 1:** The integrated renal effects of glucocorticoids. The effects of glucocorticoids on renal haemodynamics and tubular transport function can oppose one another. This is discussed further in the main text

**The effects of glucocorticoids on integrated renal function haemodynamic**
↑ renal blood flow
variable effect on renal vascular resistance
↑ filtration fraction
↑ glomerular filtration rate
**water and electrolyte metabolism**
diuresis
natriuresis or antinatriuresis – see main text
plasma volume contraction (or sometimes volume expansion – see main text)
kaliuresis
↑ renal acid excretion and metabolic alkalosis
phosphaturia
↑ amino acid transport
↑ sulphate transport
**intermediate metabolism within the kidney**
luconeogenesis
ammoniagenesis

**Figure 1:**
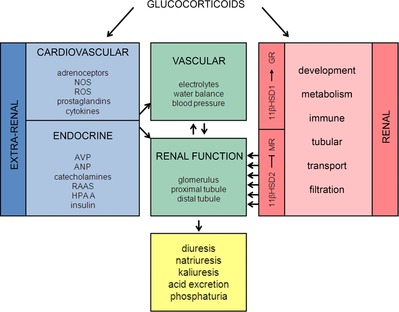
Model of glucocorticoid effects on integrated renal function Glucocorticoids act pervasively and therefore their net effect on renal function is determined by extrarenal, haemodynamic and renal tubular factors. The local action of glucocorticoids within the kidney is subject to fine-tuning by the 11βHSD isoforms. The net result is usually a natriuresis, but under some conditions antinatriuresis predominates (see main text). ANP, atrial natriuretic peptide; AVP, arginine vasopressin; GR, glucocorticoid receptor; 11βHSD1/2, 11β-hydroxysteroid dehydrogenase 1/2; HPAA, hypothalamic-pituitary-adrenal axis; NOS, nitric oxide synthase; RAAS, renin–angiotensin–aldosterone system; ROS, reactive oxygen species.

The glucocorticoid receptor (GR) is ubiquitously expressed and systemic administration of glucocorticoids therefore changes many variables, including intermediary metabolism, cardiac output and systemic vascular resistance. This integrated response to glucocorticoids is clinically relevant but it is challenging to identify primary renal events (i.e. those occurring as a direct result of glucocorticoid signalling within the kidney) from secondary responses that are indirect and often countervailing. Thus, in some circumstances glucocorticoids promote renal Na^+^ retention. This is particularly evident for endogenous glucocorticoids and reflects activation of both GR and mineralocorticoid receptors (MR) in the renal tubule.

In other cases, glucocorticoids – particularly synthetic compounds – induce a powerful natriuresis (Table 1). The conventional explanation for this phenomenon is that the haemodynamic actions of glucocorticoids impair autoregulation, increase glomerular filtration rate (GFR) and, despite the best efforts of glomerulotubular balance, promote natriuresis and kaliuresis. The mechanisms that underpin these haemodynamic effects are not fully defined. Endogenous glucocorticoids certainly exert permissive effects that help maintain both renal blood flow (RBF) and GFR: both are reduced in adrenal insufficiency. Such underperfusion does not relate exclusively to hypotension as RBF is restored by steroid replacement but not by volume replacement alone (Mangos *et al*. 2003). The effect on renal haemodynamics of exogenous glucocorticoids is more complex and the mechanisms not resolved. Micropuncture evidence in rats found that prednisolone increased single nephron GFR due to dilatation of the glomerular arterioles, with the ultrafiltration coefficient and Starling forces across the glomerular capillary being unaffected (Baylis *et al*. 1990). Similar data were obtained in dogs (Hall *et al*. 1980). However, in humans, the glucocorticoid-induced increase in GFR must reflect an increased filtration fraction as RBF remains stable or even falls, causing increased renal vascular resistance (Connell *et al*. 1987). It is not clear what accounts for these differences but one possibility is differential sensitivity to the catabolic effect of synthetic glucocorticoids as increased renal delivery of amino acids can directly increase RBF (Baylis *et al*. 1990).

The second confounding phenomenon is the capacity for glucocorticoids to activate MR, exerting effects often antagonistic to their haemodynamic actions. For example, dogs infused with noradrenaline and adrenocorticotrophic hormone (ACTH) become hypertensive and enter a negative Na^+^ and water balance. However, if the renal perfusion pressure is servo-controlled, hypertension is accompanied by Na^+^ retention (Woods *et al*. 1988). Similarly, chronic ACTH infusion into mice increases Na^+^ excretion (Dunbar *et al*. 2010) despite the fact that activation of MR and GR promotes Na^+^ reabsorption in the distal tubule (Bailey *et al*. 2009). These data underscore the dualistic effect of glucocorticoids, with the direct action on the renal tubules being over-ridden by haemodynamic processes that cause an increase in net urinary Na^+^ excretion.

Why do glucocorticoids exert these countervailing influences? Glucocorticoids induce a catabolic effect on systemic metabolism, promoting the conversion of protein, glycogen and triglyceride stores to amino acids, glucose and free fatty acids. The increase in GFR meets an increased demand for the excretion of waste products and contributes to the stress response. Teleologically, any direct stimulatory effect of glucocorticoids on tubular Na^+^ reabsorption would stabilize net excretion in the face of increased GFR, preserving salt and water balance in response to physiological stresses that threaten plasma volume. This effect is analogous to that induced by angiotensin II when activated in response to dietary Na^+^ restriction or hypovolaemia. Angiotensin II constricts the efferent arteriole to stabilize GFR in the face of reduced perfusion pressure and promotes Na^+^ retention by activation of transport proteins, including the thiazide-sensitive cotransporter (Ashek *et al*. 2012) and the epithelial Na^+^ channel (ENaC; Zaika *et al*. 2013).

However, the full extent of the physiological (and pathophysiological) role of glucocorticoids is both subtle and complex (Fig. 1). Glucocorticoids influence kidney development and the *in utero* programming of cardiovascular and renal phenotypes (Habib *et al*. 2011); they influence the pathogenesis of kidney injury (Rafiq *et al*. 2011) and they contribute to circadian variation in renal function: the glucocorticoid responsive protein, glucocorticoid-induced leucine zipper (GILZ), which features in the regulatory pathways of key Na^+^ transporters in the distal nephron (see Fig. 5), shows strong circadian oscillations in the kidney (Zuber *et al*. 2009).

**Figure 2:**
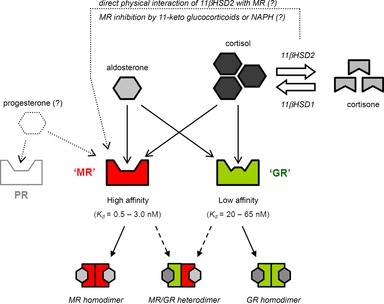
Glucocorticoid signal transduction apparatus In the kidney, glucocorticoids activate a set of receptors, GR, with low affinity and high capacity. However, they also have the capacity to activate high-affinity MR, but are prevented from doing so *in vivo* by 11βHSD2, which is expressed in mineralocorticoid-sensitive tissues. GR and MR may form heterodimers but the influence these have on Na^+^ transport is unknown. Progesterone can influence electrolyte transport by binding MR and/or its cognate receptor. GR, glucocorticoid receptor; 11βHSD1/2, 11β-hydroxysteroid dehydrogenase 1/2; MR, mineralocorticoid receptor; PR, progesterone receptor.

**Figure 3:**
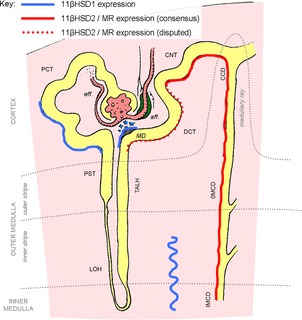
Renal sites of MR and 11βHSD expression Gluocorticoid receptors are expressed throughout the renal tubule and in the glomerulus and are not shown on this figure. See main text for details. aff., afferent; CCD, cortical collecting duct; CNT, connecting tubule; DCT, distal convoluted tubule; eff., efferent; 11βHSD1/2, 11β-hydroxysteroid dehydrogenase 1/2; IMCD, inner medullary collecting duct; LOH, loop of Henle; MD, macula densa; MR, mineralocorticoid receptor; OMCD, outer medullary collecting duct; PCT, proximal convoluted tubule; PST, proximal straight tubule; TALH, thick ascending limb of Henle's loop.

**Figure 4:**
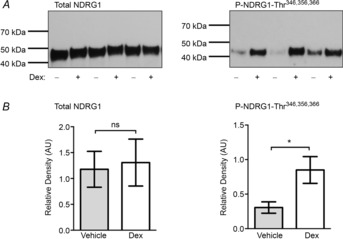
Dexamethasone increases the abundance of phosphorylated NDRG1 (P-NDRG1-Thr3^46,356,366^) in whole mouse kidney, indicative of increased SgK1 activity C57BL6 mice were treated with dexamethasone (1 mg kg^−1^) or vehicle (0.9% saline) and kidneys collected after 6 h. *A*, kidneys were probed with antibodies to the phosphorylated form of NDRG1 (P-NDRG1-Thr^346,356,366^) or total NDRG1 (T-NDRG1). NDRG1 is a substrate for SGK1 and this phosphoprotein is a surrogate indicator of SGK1 activity. *B*, densitometry analysis indicated P-NDRG1 was significantly increased in the dexamethasone-treated group but there was no change in the T-NDRG1. Data are means ± s.e.m., *n* = 6. *P < 0.05, by Student's *t* test. Dex, dexamethasone.

## Interaction between the hypothalamic–pituitary– adrenal axis and the renin–angiotensin–aldosterone system

The HPAA can influence the RAAS at both systemic and local levels. For example, ACTH excess increases the circulating mineralocorticoid ‘load’ in several ways. First, ACTH increases secretion of aldosterone by promoting cholesterol delivery to the mitochondria in the zona glomerulosa cells (Hattangady *et al*. 2012) and by enhancing CYP11B2 transcription (Takeda *et al*. 1996). Second, ACTH stimulates production of deoxycorticosterone, a weak mineralocorticoid that is physiologically significant when in excess (Mullins *et al*. 2009). The stimulatory effect of ACTH on aldosterone seems to be transient, but that on deoxycorticosterone sustained (Dunbar *et al*. 2010). Finally, ACTH may stimulate renin production in the juxtaglomerular apparatus (Oelkers *et al*. 1982), although this concept lacks much empirical support.

At a receptor level, glucocorticoids have equal, or perhaps greater, affinity for the MR than does aldosterone (Arriza *et al*. 1987). This concept is implicated in the hypokalaemia and hypertension of Cushing's syndrome and is discussed in more detail below.

## Glucocorticoid signal transduction in the renal tubule

### Pre-receptor steroid metabolism governs receptor specificity

GR and MR share a high *in vitro* affinity for both classes of steroid (Arriza *et al*. 1987), but differ in their binding kinetics. The renal MR constitutes a high-affinity, low-capacity corticosteroid-binding site (formerly designated ‘type I’), K_d_ 0.5–3 nm for both aldosterone and cortisol. GRs offer a low-affinity, high-capacity ‘type II’ site, K_d_ 20–65 nm, for both steroids. The *in vivo* specificity of the GR and MR for their cognate ligands is, at least in part, a property conferred by the pre-receptor metabolism of glucocorticoids by the 11β-hydroxysteroid dehydrogenase isozymes: type 1 (11βHSD1) converts inactive 11-keto derivatives of glucocorticoids into physiologically active cortisol (corticosterone in rodents), and type 2 (11βHSD2) catalyses the reverse reaction (Chapman *et al*. 2013). Thus 11βHSD2 confers upon MR specificity for aldosterone that is inherently lacking: cortisol activates MR whereas cortisone does not (Fig. 2). Inhibition of 11βHSD2 activity (using derivatives of glycyrrhetinic acid, the active ingredient of liquorice) promotes Na^+^ reabsorption and potassium secretion in the distal nephron (Bailey *et al*. 2001). Genetic ablation of the enzyme, as occurs in apparent mineralocorticoid excess syndrome, causes low renin hypertension and hypokalaemia due in part to increased Na^+^ reabsorption in the distal nephron (Stewart *et al*. 1996; Bailey *et al*. 2008).

Nevertheless, the physiological role of 11βHSD2 in the kidney is more complex. The pre-receptor metabolism of glucocorticoids by the enzyme will protect MR but cells of the distal tubule also express GR. It is unlikely that these receptors are physiologically redundant and their activation will be influenced by 11βHSD2-mediated metabolism of cortisol. In heterologous expression systems, 11βHSD2 appears able to influence the subcellular localization of MR, possibly through a direct physical interaction. Furthermore, cortisone blocks the interaction between aldosterone and MR, suggesting that the ‘inactive’ 11-keto-glucocorticoids generated by 11βHSD2 may act as autocrine or paracrine MR antagonists (Odermatt *et al*. 2001). NAPH generation by 11βHSD2 can also alter the intracellular redox potential, locking MR–cortisol complexes in an inactive state (Funder, 2010). Thus, glucocorticoids can bind MR but the receptor is not physiologically activated unless excess reactive oxygen species are present. This adverse redox environment is generated in the kidney of salt-sensitive animals after a period of high Na^+^ intake: glucocorticoid-induced activation of MR promotes inflammation and fibrosis (Luther *et al*. 2012).

There is also the potential for interaction with sex steroids, as progesterone acts as a partial agonist for MR and GR (Arriza *et al*. 1987). Moreover, *bona fide* progesterone receptors are also expressed in the distal nephron, where they probably participate in the regulation of solute transport. Progesterone derived from the adrenal gland promotes renal potassium retention in male and ovariectomized female potassium-depleted mice (Elabida *et al*. 2011). This probably reflects direct signalling via the progesterone receptor as activation of GR or MR would be kaliuretic. Moreover, the potassium retention was blocked by RU486, an antagonist of the progesterone receptor. RU486 also antagonizes GR but the progesterone-induced potassium retention was not associated with induction of classic GR response genes.

### Renal expression of glucocorticoid receptor, mineralocorticoid receptor and 11β-hydroxysteroid dehydrogenase isozymes

The expression patterns of MR and 11βHSD within the kidney are depicted in Fig. 3: GR is widely expressed in the kidney, with mRNA being detected in most cells. In contrast, MR and 11βHSD2 have a more restricted distribution: co-localization of these in the connecting tubule and principal cells of the collecting duct defines the ‘aldosterone-sensitive distal nephron’ (ASDN). The expression of 11βHSD2 in the distal convoluted tubule (DCT) is less certain and it is probable that the enzyme is not expressed at high levels, if at all (Bostanjoglo *et al*. 1998; Campean *et al*. 2001). Although DCT cells express both GR and MR and are responsive to both corticosteroids, the conventional model of the ASDN does not apply in this segment. This is an important concept as the DCT reabsorbs more of the filtered Na^+^ load (∼7%) than does the collecting duct (<5%). Abnormal glucocorticoid status could, via activation of the thiazide-sensitive transporter in the DCT, imperil Na^+^ and blood pressure homeostasis.

Several groups have proposed that 11βHSD2 abundance decreases along a gradient as one moves proximally from the cortical collecting duct, through the connecting tubule, to DCT. This has led to speculation that there may be an ‘ASDN proper’ in which aldosterone dominates regulation of Na^+^ transport through MR, and an intermediate segment expressing low levels of 11βHSD2 in which MR is activated by aldosterone under basal conditions and/or by glucocorticoids if they are present in excess, during activation of the HPAA or at certain periods during the circadian cycle (Gaeggeler *et al*. 2005). It is also possible that 11βHSD2 expression/activity is physiologically regulated by Na^+^ and K^+^ status (Thompson *et al*. 2000): DCT and the connecting tubule are plastic, homeostatically responsive epithelia capable of rapid remodelling of the molecular apparatus for Na^+^ transport.

### 11β-Hydroxysteroid dehydrogenase 1 and the glucocorticoid-amplified proximal nephron

11βHSD1 is located in the S3 proximal tubule (Fig. 3) and macula densa (Gong *et al*. 2008; Odermatt & Kratschmar, 2012). It has also been detected in the interstitial cells of the renal medulla (Castello *et al*. 1989; Rundle *et al*. 1989). As these cells lack the hexose-6-phosphate dehydrogenase and cannot generate NADPH, 11βHSD1 may therefore act as a dehydrogenase here, catalysing the same reaction as 11βHSD2 (Gomez-Sanchez *et al*. 2008). The urinary steroid profile following siRNA knockdown of medullary 11βHSD1 suggests that this is not correct and 11βHSD1 acts predominantly as a reductase (Liu *et al*. 2008). This leads to the concept of a ‘glucocorticoid-amplified proximal nephron’ and detailed physiological examination of 11βHSD1 activity in the renal tubule is required. Data are surprisingly scarce but downregulation of renal 11βHSD1 is an adaptive response to either salt loading or increased blood pressure (Dunbar *et al*. 2010). Failure to transcriptionally repress the encoding gene, *hsd11b1*, contributes to the pathogenesis of salt-sensitive hypertension. This phenomenon was demonstrated in innovative studies using the Dahl salt-sensitive (DSS) rat and a consomic control strain (DSS-13^BN^) with attenuated salt sensitivity of blood pressure. Renal medullary expression of 11βHSD1 was downregulated in response to high dietary salt in DSS-13^BN^ but not in DSS rats. The authors hypothesized that failure to downregulate 11βHSD1 contributed to renal Na^+^ retention and hypertension. To test this hypothesis, knockdown of 11βHSD1 expression/activity was induced by injecting siRNA into the renal medulla *in vivo*: *hsd11b1* knockdown attenuated salt-sensitive hypertension in the DSS rats (Liu *et al*. 2008). The molecular mechanism underpinning the rescue of salt-sensitive blood pressure was not determined. It may be that 11βHSD1 knockdown had a direct impact on Na^+^ transport processes in the medullary tubular epithelium (i.e. on NKCC2 function in the thick ascending limb of Henle's loop). There is no consensus in the literature concerning the effect of glucocorticoids on NKCC2 activity, with both transcriptional repression of *slc12a1* (Bailey *et al*. 2009) and increased abundance of the protein (Frindt & Palmer, 2012) being reported. Nevertheless, *hsd11b1* knockdown in the renal medulla resulted in a reduction in the concentration of corticosterone in the urine. This raises the possibility that 11βHSD1 in the interstitial cells of the medulla exerts a paracrine effect on transport processes in the distal nephron by altering the concentration of active glucocorticoids in the downstream tubular fluid and/or peritubular capillaries.

### Glucocorticoid receptor in the distal nephron

Classical studies of receptor–ligand interactions in collecting duct cells *in vitro* demonstrate that mineralocorticoids can bind to the GR. Indeed, binding assays indicate that physiological concentrations of aldosterone would induce a low level of GR occupancy, but the biological significance of this is not clear (Gaeggeler *et al*. 2005). An interaction between aldosterone status and the GR has been demonstrated in mouse and rat kidneys (Ackermann *et al*. 2010), using nuclear translocation as a proxy for receptor activation. Contrary to our understanding of the ASDN, suppression of aldosterone by dietary NaCl loading resulted in a reduction in the nuclear localization of GR in the ASDN whereas MR localization was not affected. Conversely, adrenalectomy resulted in the loss of nuclear MR and GR in all nephron segments. The nuclear MR signal was restored in all nephron segments by physiological doses of corticosterone. The GR signal was restored in most nephron segments but not in the ASDN. Furthermore, in a colonic cell line expressing both receptors (an unusual phenomenon in epithelial cell lines), GR activation did not itself induce ENaC but was a prerequisite for the full MR-mediated response to aldosterone (Bergann *et al*. 2011). Similar findings have been reported in neuronal cell lines (Tsugita *et al*. 2009). The interaction between the receptors is not understood but GR may be *sine qua non* for formation of the MR/MR homodimer or may even form a heterodimer with MR (Fig. 2), as is suggested by FRET microscopy (Nishi *et al*. 2004).

These data challenge our conventional view of the steroid control of Na^+^ transport in the ASDN and the consequences for Na^+^ transport are not clear. If glucocorticoids, via GR, physiologically regulate MR, an important homeostatic role for 11βHSD2 may be to control intracellular glucocorticoid concentration and thereby govern GR activation.

## Signal transduction downstream of steroid receptor activation

Activated steroid receptors translocate to the nucleus, where they act as transcription factors. There is considerable overlap in the GR and MR response genes, providing an additional mechanism through which both classes of steroid can activate a common set of biological effector pathways.

### Genomic responses to glucocorticoids: well-characterized pathways

GR activation in the collecting duct stimulates the transcription of Sgk1 and GILZ (Muller *et al*. 2003; Nguyen Dinh Cat *et al*. 2009). However, the role of Sgk1 in the renal response to glucocorticoids *in vivo* remains obscure. Dexamethasone increases the abundance of Sgk1 transcripts in whole kidney homogenates. Our data indicate that Sgk1 is physiologically active: dexamethasone increases the phosphorylation of the Sgk1 target NDRG1 (Fig. 4). Dexamethasone does not, however, increase Sgk1 expression in isolated cortical collecting ducts (Muller *et al*. 2003); Sgk1 expression in the distal renal tubule was not altered in response to overexpression of GR in the collecting duct (Nguyen Dinh Cat *et al*. 2009). These observations suggest that whereas Sgk1 participates in the response to glucocorticoids in some kidney cells, it does not do so in the ASDN, where some (yet unknown) mechanisms preserve Sgk1 as an aldosterone-responsive gene. Dexamethasone upregulates NHE3 activity in cultured renal cells in an Sgk1-dependent fashion, providing *in vitro* evidence that Sgk1-dependent pathways may participate in glucocorticoid-regulated solute transport in the proximal nephron (Wang *et al*. 2007).

Both Sgk1 and GILZ are also classic MR response genes (Fig. 5). The Sgk1-Nedd4–2-ENaC pathway provides the canonical mechanism whereby aldosterone stimulates Na^+^ reabsorption in the principal cell (Snyder *et al*. 2002); the Sgk1-Nedd4–2 pathway also operates in the DCT to stimulate NCC (Arroyo *et al*. 2011). In cultured collecting duct (mpkCCD) cells, GILZ participates in the regulation of ENaC activity by aldosterone (Soundararajan *et al*. 2005).

**Figure 5:**
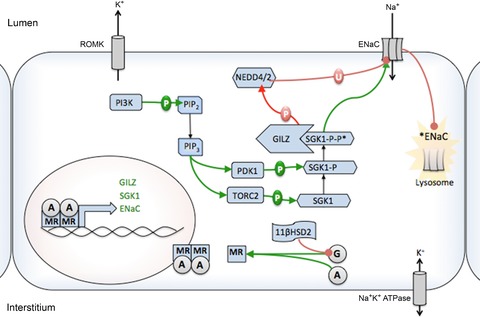
Paradigm of mineralocorticoid signalling: regulation of ENaC in the principal cell Aldosterone regulates ENaC through MR-binding dependent promotion of SGK-1 expression and ENaCα expression. Two phosphorylation steps, mediated by TORC2 and PDK1, activate SGK1 (SGK1-P-P*). These kinases are activated by the PI3K system. SGK1 phosphorylates Nedd4/2, which is an ubiquitin ligase enzyme that binds ENaC and marks it for withdrawal from the apical membrane and subsequent degradation. SGK1-dependent phosphorylation inhibits Nedd4/2-ENaC binding and promotes the maintenance of ENaC in the membrane. Glucocorticoids are prevented from activating MR in the presence of high levels of 11βHSD2. GILZ expression is also increased upon aldosterone stimulation and is thought to stabilize SGK1. Red connectors represent inhibition through phosphorylation or ubiquitinylation, while green arrows indicate stimulation by phosphorylation or otherwise. Black arrows indicate movement of ions or change to phosphorylated state. A, aldosterone; ENaC, epithelial Na^+^ channel; G, glucocorticoids; 11βHSD1/2, 11β-hydroxysteroid dehydrogenase 1/2; MR, mineralocorticoid receptor; P, phosphorylation; U, ubiquitinylation.

**Figure 6:**
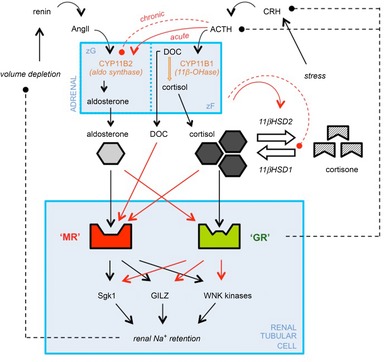
Interactions between the RAAS and the HPAA Dashed arrows (with filled circles at the head) represent negative feedback loops. Red arrows are potential sites of crosstalk between the RAAS and the HPAA. Interactions arise at multiple levels: pre-receptor (changes in ligand availability), receptor (receptor–ligand promiscuity) and post-receptor (common second messenger systems). HPAA activation can increase the activity of 11βHSD2 and inhibit 11βHSD1. The other potential routes of crosstalk are discussed in the main text. ACTH, adrenocorticotrophic hormone; AngII, angiotensin II; CRH, corticotrophin releasing hormone; DOC, deoxycorticosterone; HPAA, hypothalamic–pituitary–adrenal axis; 11βHSD1/2, 11β-hydroxysteroid dehydrogenase 1/2; MR, mineralocorticoid receptor; RAAS, renin–angiotensin–aldosterone system.

### Genomic responses to glucocorticoids: an unbiased approach

We have made a systematic attempt to identify the renal transcriptional response to glucocorticoids in mice exposed to 12 days of exogenous ACTH. mRNA prepared from whole kidneys was hybridized with an Affymetrix GeneChip (Santa Clara, CA, USA), and the results subjected to a pathway analysis (Dunbar *et al*. 2010). This model predominantly reflects glucocorticoid-mediated signalling, the stimulation by ACTH of aldosterone production (*vide supra*) being transient (Dunbar *et al*. 2010). A large number of genes were differentially regulated, including known targets such as Sgk1 as well as novel gene pathways concerned with organic anion/cation transport, vitamin D, calcium and xenobiotic metabolism.

### The WNK-SPAK cascade

The WNK-SPAK kinase network acts as a master regulator of electrolyte transport in the distal renal tubule, and can transduce mineralocorticoid signals (Hoorn *et al*. 2011). Sgk1 phosphorylates WNK4 at ser^1169^, thus relieving inhibition by WNK4 of downstream targets, including NCC, ENaC and ROMK (Ring *et al*. 2007; Rozansky *et al*. 2009).

However, GR can also regulate WNK signalling. WNK4 expression is reduced when GR is overexpressed in the collecting duct (Nguyen Dinh Cat *et al*. 2009) and GR negatively regulates WNK4 transcription in mpkDCT cells and in the murine DCT *in vivo* (Mu *et al*. 2011) (*vide infra*). Basal WNK4 mRNA expression is higher in mice lacking GR in the distal nephron, suggesting that endogenous glucocorticoids exert a tonic antinatriuretic effect through their effects on WNK signalling (Mu *et al*. 2011).

## Glucocorticoid effects on tubule Na^+^ transport

The systemic administration of glucocorticoids induces effects on solute transport processes along the length of the renal tubule. For example, dexamethasone treatment in rats causes an increase in the abundance of NHE3, NCC, NKCC2, the full-length isoform of α-ENaC and the cleaved isoform of γ-ENaC in whole kidney homogenates (Frindt & Palmer, 2012). Glucocorticoids influence cellular morphology and proliferation in the distal renal tubule, causing amplification of the basolateral membranes of the principal cells in the cortical collecting tubules in the rabbit (Wade *et al*. 1979). However, these studies are unable to discriminate between the specific effects of glucocorticoid signalling in renal tubular cells and a ‘passive’ response to glucocorticoid-induced changes in systemic haemodynamics and/or intermediate metabolism.

## Glucocorticoid effects in the proximal tubule

### Regulation of NHE3 and Na^+^-P_i_ cotransporter 2

In the rat glucocorticoids stimulate Na^+^ bicarbonate reabsorption by activating NHE3 (Zallocchi *et al*. 2003) and suppress sodium phosphate cotransport by Na^+^-P_i_ cotransporter 2 (Loffing *et al*. 1998). These events contribute to glucocorticoid-induced increases in the renal excretion of acid and phosphate (Table 1).

## Glucocorticoid effects in the distal convoluted tubule

Glucocorticoids stimulate NaCl reabsorption in the DCT. The underlying molecular mechanisms show the DCT to be a site at which several key natritropic signals interact. In a mouse model of salt-sensitive hypertension with sympathetic activation, an epigenetic interaction between adrenergic and glucocorticoid signalling is indicated. This effect is mediated by the WNK kinases and results in the regulation of NCC expression, phosphorylation and transport activity (Mu *et al*. 2011). β-adrenergic stimulation activated NCC by suppressing WNK4 expression through a GR-dependent mechanism. Activated β2-adrenoreceptors induced histone acetylation at negative glucocorticoid response elements in the WNK4 promoter, enhancing GR binding. There was corresponding negative regulation of Wnk4 by GR in the DCT *in vivo*: adrenalectomy or a GR antagonist abolished the inhibitory effect of noradrenaline on Wnk4 mRNA expression (and this was restored by dexamethasone in the case of adrenalectomy).

As WNK4 is itself a negative regulator of NCC activity, these findings support a model in which GR activation in the DCT tonically stimulates NaCl reabsorption. Consistent with this, basal Wnk4 mRNA abundance was higher in mice lacking GR in the distal nephron.

## Glucocorticoid effects in the aldosterone-sensitive distal nephron: *in vitro* and *in vivo* data

The ability of glucocorticoids to stimulate electrogenic Na^+^ transport in the collecting ducts has been demonstrated *in vitro* using cultured cell lines that faithfully maintain many of the *in vivo* characteristics of principal cells (Naray-Fejes-Toth & Fejes-Toth, 1990; Laplace *et al*. 1992; Bens *et al*. 1999; Gaeggeler *et al*. 2005). In mCCD_cl1_ cells, corticosterone stimulates amiloride-sensitive transport even in the presence of intact 11βHSD2 (Gaeggeler *et al*. 2005), albeit at higher concentrations than aldosterone (K_½_ = 18 nm for corticosterone; 0.52 nm for aldosterone). High concentrations of dexamethasone stimulate this current via an RU486-sensitive pathway, suggesting that this is a consequence – at least in part – of GR activation. The mechanisms whereby GR stimulates Na^+^ transport in the collecting duct remain to be established: regulation of endothelin-1 expression has been implicated *in vitro* (Stow *et al*. 2012).

### Glucocorticoid effects on Na^+^ transport in the ‘aldosterone-sensitive distal nephron’ *in vivo*

Despite the robust *in vitro* data, a direct demonstration that glucocorticoids physiologically activate ENaC *in vivo* has been lacking. Seven days of treatment with dexamethasone increased the abundance of the full-length isoform of α-ENaC in rat kidney, but had no effect on electrogenic Na^+^ transport in split open collecting ducts (Frindt & Palmer, 2012). Mice heterozygotes for a null mutation in *Hsd11b2* (the gene encoding 11βHSD2) have salt-sensitive blood pressure associated with elevated levels of circulating glucocorticoids (Bailey *et al*. 2011). Salt loading induced hypokalaemia in hsd11b2^+/−^ mice, and the trans-tubular potassium gradient >7 indicated enhanced ‘mineralocorticoid’ activity in the distal nephron. The salt sensitivity and hypokalaemia were both corrected by GR antagonism but not by MR antagonism, suggesting that GR activation might exert a pathophysiological role in the ASDN.

The MR knockout mouse provides further evidence of glucocorticoids exerting mineralocorticoid effects in the distal nephron. Global constitutive knockout in MR is fatal; the animals die from excessive urinary solute losses at about day 10, indicating that GR is not able to completely compensate for the lack of MR (Berger *et al*. 1998). However, glucocorticoids are capable of a partial compensation: triamcinolone treatment enhanced ENaC expression and activity in salt-supplemented MR knockout mice (Schulz-Baldes *et al*. 2001).

The direct effects of GR signalling in the distal nephron has been investigated using two transgenic mouse models: Ksp-Cre^+^ GR^loxP/loxP^ mice, in which GR was specifically and constitutively deleted in the AQP2-positive distal nephron (Goodwin *et al*. 2010), and Hoxb7-tetON2-hGR mice in which GR was conditionally overexpressed in cells of collecting duct lineage (Nguyen Dinh Cat *et al*. 2009). Loss of GR from the distal nephron had no effect on the response to chronic dexamethasone, which induced a rise in blood pressure and a natriuresis that was indistinguishable from that in wild-type mice (Goodwin *et al*. 2010). However, basal blood pressure was higher in Ksp-Cre^+^ GR^loxP/loxP^ mice, raising the possibility that GR activity in the ASDN participates in blood pressure homeostasis in the ‘normal healthy’ adult. This being a constitutive knockout, a developmental effect cannot be discounted. Overexpression of GR in the distal nephron had no effect on net urinary Na^+^ or K^+^ excretion, although there was a reduction in urinary aldosterone indicative of compensatory RAAS inhibition (Nguyen Dinh Cat *et al*. 2009). There was a clear transcriptional response to GR overexpression (discussed above). Taken together, these data suggest that GR-mediated signalling may modulate Na^+^ transport in the collecting duct, but the regulation of net renal Na^+^ excretion is physiologically dominated by the RAAS. Moreover, it is possible that some of the effects of mineralocorticoids in the distal nephron may be mediated independently of MR and GR. In rats chronically infused with aldosterone, combined MR and GR blockade did not prevent the increase in ENaC protein abundance and trafficking to the apical cell membrane (Nielsen *et al*. 2007). The physiological effect of this is uncertain: spironolactone did rescue the hypokalaemia and prevented the increase in Na^+^/K^+^-ATPase abundance, suggesting that electrogenic Na^+^ transport in the principal cell was reduced, despite the continued presence of ENaC in the apical membrane.

## Implications for hypertension and salt sensitivity in humans

The renal tubules possess the molecular apparatus requisite for glucocorticoid-responsive Na^+^ transport. The expression of 11βHSD1 and 11βHSD2 will influence these processes by regulating local glucocorticoid concentrations. Several lines of evidence challenge the conventional role of renal 11βHSD2 as a mere enzymatic guardian of the MR. Equally compelling, is evidence showing stimulation by glucocorticoids of Na^+^ reabsorption at several sites along the nephron. More provocatively, experiments in cell lines and in native kidney indicate that GR has an important permissive role for aldosterone signalling at MR. The HPAA is thus able to influence renal Na^+^ excretion at multiple levels (Fig. 6) – and such regulation escapes the negative feedback mechanisms inherent within the RAAS.

However, is this relevant for human health? Many of the mechanisms whereby glucocorticoids cause hypertension reside in the vasculature, and central and autonomic nervous systems, but there is a clear contribution from an antinatriuretic effect in the renal tubules. This is clinically important: salt-sensitive humans have an enhanced stress-induced activation of the HPAA (Weber *et al*. 2008) and attenuated glucocorticoid clearance (Kerstens *et al*. 2003). Glucocorticoids stimulate electrogenic Na^+^ transport in humans when present in excess, as in Cushing's syndrome. We do not know, however, if this contributes to hypertension in states of moderate glucocorticoid excess or in the metabolic syndrome, where the tissue availability of active glucocorticoid is enhanced (Pereira *et al*. 2012). *Hsd11b2* gene polymorphisms are associated with salt sensitivity in blood pressure in normotensive and hypertensive subjects, suggesting that glucocorticoids can breach the 11βHSD2 barrier even when they are not present in vast excess.

The molecular pathways whereby glucocorticoids contribute to salt-sensitive hypertension have been elucidated in mice. These studies provide a mechanistic explanation for the long-recognized ability of combined glucocorticoid and adrenergic stimulation to exert tubular antinatriuretic effects. Mechanisms in the distal nephron predominate: the NCC is activated during sympathetic stimulation (Mu *et al*. 2011) and ENaC is increased in 11βHSD2 heterozygotes (Craigie *et al*. 2012). These pathways are attractive therapeutic targets to ameliorate the salt-sensitive hypertension associated with chronic stress and other states of sympathetic and HPAA activation in humans. There is also a pressing clinical need to develop a mechanistic understanding of the effects of renal 11βHSD1 activity on Na^+^ transport. Systemic 11βHSD1 inhibitors are in development for use as modifiers of cardiovascular risk in obesity, type 2 diabetes mellitus and the metabolic syndrome (Hughes *et al*. 2008; Hadoke *et al*. 2009), conditions in which salt-sensitive hypertension is prevalent (Hall, 2003; Fujita, 2010). One potential side-benefit of 11βHSD1 inhibition in such cases might be an improvement in salt sensitivity because of diminished active glucocorticoid generation in the renal medulla. An antihypertensive effect of 11βHSD1 inhibitors has recently been reported in a clinical trial and in the spontaneously hypertensive rat (Bauman *et al*. 2013).

## Conclusion

Conditions associated with modest elevations in circulating glucocorticoids are common. Moreover, the widespread therapeutic use of GR agonists may flatten the dynamic regulation of the HPAA with deleterious consequences for renal Na^+^ homeostasis. Glucocorticoids regulate Na^+^ transport in the proximal and distal renal tubule and, in particular, can stimulate Na^+^ reabsorption in the post-macular segments. 11βHSD2 activity is low in the DCT, which is emerging as a critical site for the regulation of renal Na^+^ excretion by various signalling pathways, including glucocorticoids. These effects have implications for human health and disease, with the potential to contribute to the pathogenesis of Na^+^-sensitive hypertension in the metabolic syndrome and in chronic stress.
